# On-site single pollen metabolomics reveals varietal differences in phosphatidylinositol synthesis under heat stress conditions in rice

**DOI:** 10.1038/s41598-020-58869-9

**Published:** 2020-02-06

**Authors:** Hiroshi Wada, Yuto Hatakeyama, Taiken Nakashima, Hiroshi Nonami, Rosa Erra-Balsells, Makoto Hakata, Keisuke Nakata, Kenzo Hiraoka, Yayoi Onda, Hiroshi Nakano

**Affiliations:** 1 Kyushu Okinawa Agricultural Research Center, National Agriculture and Food Research Organization, 496 Izumi, Chikugo, Fukuoka 833-0041 Japan; 2 Faculty of Agriculture, Hokkaido University, Kita-9 Nishi-9, Kita-Ku, Sapporo 060-8589 Japan; 3 Graduate School of Agriculture, Ehime University, 3-5-7 Tarumi, Matsuyama, 790-8566 Japan; 4 Department of Organic Chemistry, University of Buenos Aires, Buenos Aires, 1428 Argentina; 5 Clean Energy Research Center, The University of Yamanashi, 4-3-11 Takeda, Kofu, Yamanashi 400-8511 Japan

**Keywords:** Pollen, Pollination, Plant physiology, Plant stress responses, Heat, Metabolomics

## Abstract

Although a loss of healthy pollen grains induced by metabolic heat responses has been indicated to be a major cause of heat-induced spikelet sterility under global climate change, to date detailed information at pollen level has been lacking due to the technical limitations. In this study, we used picolitre pressure-probe-electrospray-ionization mass spectrometry (picoPPESI-MS) to directly determine the metabolites in heat-treated single mature pollen grains in two cultivars, heat-tolerant cultivar, N22 and heat-sensitive cultivar, Koshihikari. Heat-induced spikelet fertility in N22 and Koshihikari was 90.0% and 46.8%, respectively. While no treatment difference in *in vitro* pollen viability was observed in each cultivar, contrasting varietal differences in phosphatidylinositol (PI)(34:3) have been detected in mature pollen, together with other 106 metabolites. Greater PI content was detected in N22 pollen regardless of the treatment, but not for Koshihikari pollen. In contrast, there was little detection for phosphoinositide in the single mature pollen grains in both cultivars. Our findings indicate that picoPPESI-MS analysis can efficiently identify the metabolites in intact single pollen. Since PI is a precursor of phosphoinositide that induces multiple signaling for pollen germination and tube growth, the active synthesis of PI(34:3) prior to germination may be closely associated with sustaining spikelet fertility even at high temperatures.

## Introduction

In rice, extremely high temperature conditions at flowering have caused widespread yield instability across many production areas, due to heat-induced spikelet sterility under global warming^1–3^. It has been accepted that exposure to temperatures higher than 35 °C at flowering induces spikelet sterility, leading to yield loss based on growth chamber experiments^4^. Many researchers have put into efforts to study the underlying mechanisms behind spikelet sterility. It has been reported that the ability of pistil to be fertilized remained unaffected when exposed to high temperatures below 41 °C, and a loss of heat-induced fertility was mostly attributed to decreased pollen viability and disturbed pollen shedding, resulting in the reduction in germinated pollen grains on a stigma^4^. A decline in population of healthy pollen grains has gained attention as a cause of spikelet sterility^5^, as well as anther indehiscence^4,6^. Although multiple quantitative trait loci (QTL) have been identified for heat tolerance at flowering^7^, information is still limited and no superior heat-tolerant cultivars at flowering have been developed, at least in Japan^8^. For reasons of convenience and rapidness, *in vitro* pollen viability has been widely determined by using iodine staining for screening in rice breeding; however, this method has been recently called into question^1^. As the heat risk is expected to intensify with respect to global climate change, it is highly desirable to speed up rice breeding through identification of more reliable molecular marker(s). However, it seems likely that much less attempts have been conducted in the view of pollen physiology in rice.

Pollen grains are male microgametophytes, consisting of the vegetative and the  generative cells. The generative cell divides to form twin sperm cells, and the resulting male gametophyte is called the tricellular pollen^9^. Upon pollen germination, the pollen tube extends and grows down toward the ovule, and two sperm nuclei enter the female gametophyte and participate in a double fertilization process. According to the early microscopic observation^10^, the developmental stage of rice pollen before flowering corresponds to the mature tricellular pollen, where starch degradation progressively occurs prior to anther dehiscence. Once the anther has dehisced, pollen falls on to the stigma, followed by pollen hydration and germination on the stigma^11^. Rice-like anemophilous pollen accumulate mostly starch, lipid, and proteins required for germination and pollen tube growth. During the last period of development, pollen accumulates two types of lipids, the neutral lipids stored in oil lipids and the polar lipids in the form of densely packed membranes^12^. The neutral lipids, including triacylglycerol, derived from the diploid tapetal cells, were closely associated with both desiccation tolerance and pollen adhesion to the stigma. In contrast, the polar lipids including membrane-associated phospholipids formed by the haploid vegetative cells would be used for pollen tube growth^12,13^.

There is accumulating evidence that inositol-containing lipids, phosphoinositide species can promote pollen hydration, germination and tip growth through multiple signaling^12,14–16^. One of the phospholipids, called phosphatidylinositol (PI), is known to be generated by catalyzing the transfer of free inositol to the backbone of a glycerophospholipid through the activation of PI synthase (EC 2.7.8.11) in the endoplasmic reticulum as the phosphoinositide precursor. In sorghum, it has been reported that changes in pollen phospholipids accompanied by an increase in reactive oxygen species (ROS) at high night temperatures may be closely associated with the reduction in pollen function^17^. By using lipidome analysis in pooled wheat pollen, it has been shown that C34:3 and C36:6 species are predominant phospholipids^17^, although the exact chemical composition of the predominant phospholipids has not been identified, implying the requirement of high-resolution cell metabolomics. So far, little is known about the possible changes in phospholipid biosynthesis in rice pollen in response to heat. However, if rice pollen exhibits similar heat responses, then PI synthesis may be the prerequisite for turning on the signaling even under heat conditions. In this work, we have hypothesized that PI biosynthesis in the mature pollen is closely associated with rice heat tolerance for spikelet sterility. Given that lipid metabolism is highly sensitive to temperature change, determining cytosolic metabolite(s) in the single pollen grain being exposed to heat would be ideal but challenging. Furthermore, methods to do the required analysis under heat-like temperature stress were not available until recently.

We have used a newly developed *on-site cell-specific analysis* (see Fig. S1 in Wada *et al*. 2019)^18^ to make this analysis possible. This analytical method consists of single cell metabolomics, called ‘picolitre pressure-probe-electrospray-ionization mass spectrometry (picoPPESI-MS)’^19^ and accurate environmental control. The picoPPESI-MS technique has been successfully applied to several cell-specific research in different plant tissues, such as tomato trichomes^19^, rice endosperms^18^, and developing xylems in Norway spruce^20^. In this study, we have directly inserted the tip of a finely tapered quartz capillary tip into the developing pollen grains in intact plants growing under controlled high temperature environments to identify the internal metabolites. Two varieties, heat-tolerant cultivar, N22^21^ and heat-sensitive cultivar, Koshihikari^22^, have been provided to test our hypothesis. Here, we show that there was a clear varietal difference in PI content in developing ungerminated pollen, which may be closely associated with heat tolerance and contribute to the steady spikelet fertility. Several heat-induced metabolic changes in PI-related pathway will be discussed.

## Results

### Spikelet fertility under heat conditions

**Figure 1:**
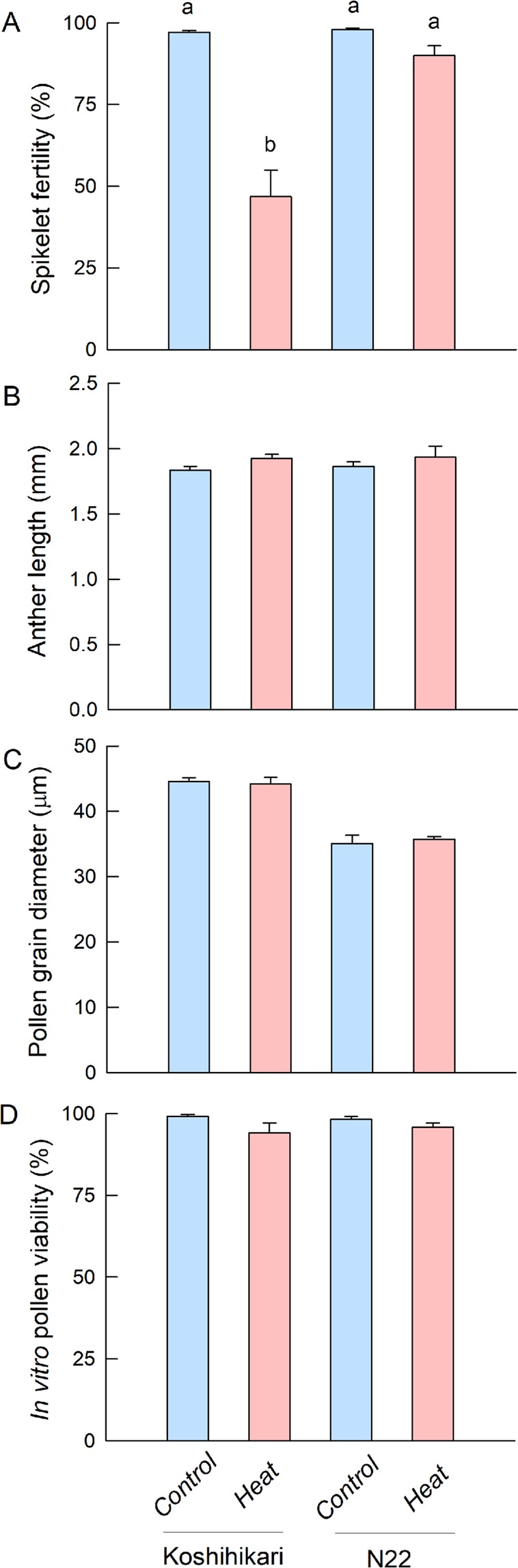
Spikelet fertility (**A**), anther longitudinal length (**B**), pollen grain diameter (**C**), and *in vitro* pollen viability (**D**) in Koshihikari and N22 grown under high temperature conditions for 48 h after heading in the growth chambers. Data in (**A,B,D**) are means (±SE) of 3–4 replicates. Data in C are means (±SE) of 3–6 replicates. Means followed by different letters indicate significant differences between treatments according to two-way ANOVA followed by Tukey’s test (*P* ≤ 0.05).

Two-way ANOVA revealed significant cultivar and heat temperature effects as well as a significant effect of their interaction (Table 1). Spikelet fertility in the Koshihikari control group was ca. 96%, although there was a remarkable decline in fertility of Koshihikari spikelets when exposed to heat conditions, reaching 46.8% (Fig. 1A). In contrast, spikelet fertility in N22 control declined slightly under heat conditions, but with no significant treatment difference (Fig. 1A).

**Table 1:** Two-way analysis of variance (ANOVA) of the effects of cultivar, heat treatment and their interaction on spikelet fertility, anther length, pollen grain diameter, and *in vitro* pollen viability in growth chamber-grown rice.

	Cultivar	Treatment	Cultivar × treatment
Spikelet fertility	<0.001	<0.001	<0.001
Anther length	0.726	0.168	0.882
Pollen grain diameter	<0.001	0.885	0.546
*In vitro* pollen viability	0.821	0.061	0.485

The F-value probabilities at 95% confidence are indicated.

### Anther length, pollen grain morphology and *in vitro* viability under heat conditions

There were no varietal and treatment effects and no significant interaction in anther length (Fig. 1B, Table 1). According to two-way ANOVA, a significant cultivar effect was observed in the pollen grain diameter, but not for both treatment effect and the interaction (Table 1). The diameter in Koshihikari pollen grains was 44 μm on average, whereas the mean diameter of N22 pollen grains was 35 μm, slightly smaller than that of Koshihikari (Fig. 1C). The number of pollen grains per anther in control in Koshihikari and N22 was 1002 and 1248 on average (*n* = 3), respectively, and a significant varietal difference was observed by *t*-test (*P = *0.02). Although *in vitro* pollen viability was prone to decline under heat conditions regardless of cultivars, but with no significant cultivar and treatment effects and the significant interaction between the two factors (Fig. 1D, Table 1).

### Profiling pollen metabolites under normal conditions

**Figure 2:**
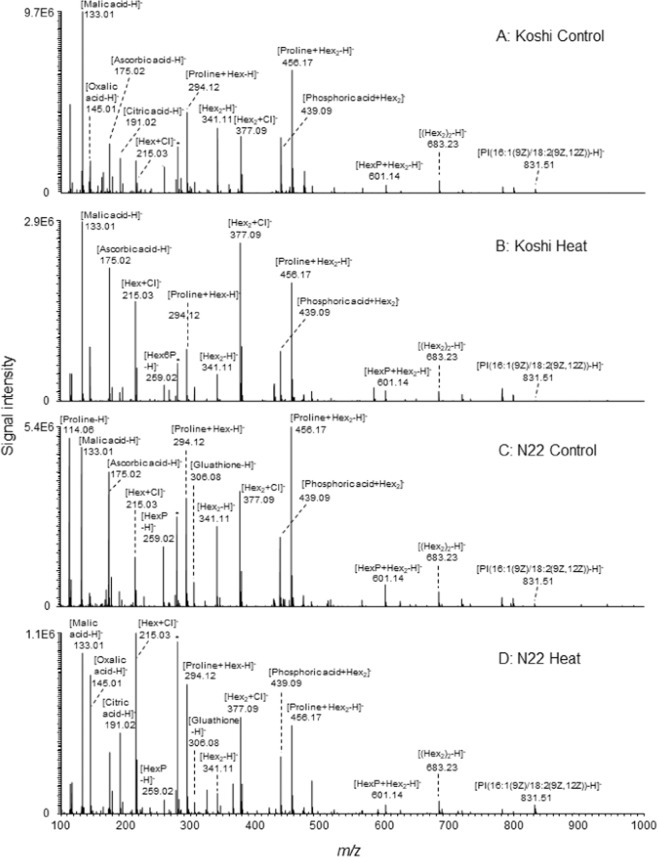
PicoPPESI mass spectra in negative ion mode obtained from the single pollen grains for the different treatments. The data are representative of repeated experiments with 8–16 pollen grains in total from 4–5 plants in each treatment. Asterisk indicates a cluster from ionic solution used.

By using the analytical method, 107 metabolites in total (133 signals in total including cluster ions), including organic acids, amino acids, carbohydrates, cell wall-related materials, and hormones, such as gibberellin A3 (*m/z* 345), trans-zeatin (*m/z* 218), and salicylic acid (*m/z* 137), have been simultaneously detected in the single pollen grains (Fig. 2A,C, Table S1). In both cultivars, the peaks of proline (*m/z* 114), ascorbic, citric, malic, and 2-oxoglutaric acids (as [M − H]^−^, M = molecular species), sugars (as [M − H]^−^ and/or [M + Cl]^−^), and sugar-organic acid clusters (as [M’ − H]^−^, M’ = summation of individual molecular mass of chemical cluster component species) were identified as major ions (Fig. 2A–D, Table S1). N22 pollen grains were prone to accumulate greater amount of succinic acid (*m/z* 117), pentose (*m/z* 149, significant at *P* > 0.08), Hex (*m/z* 179, 215), proline (*m/z* 114), and their cluster ions ([Proline+ Hex−H]^−^ (*m/z* 294), [Proline+Hex_2_−H]^−^ (*m/z* 456)), glutathione (*m/z* 306), Hex_2_ (*m/z* 377 as [M + Cl]^−^), AMP (*m/z* 346), glycerol 3-phosphate (*m/z* 171), PI(C34:3)(*m/z* 832), phosphatidylethanolamine, PE(C34:3)(*m/z* 712), phosphatidic acid, PA(C34:3)(*m/z* 669), and most fatty acids, compared with Koshihikari (Fig. 2, Table S1). In addition, HexP (*m/z* 259) and HexP_2_ (*m/z* 338) were frequently detected, but there was no or little detection for other hexose phosphates, such as HexP_3_ (*m/z* 418) and HexP_4_ (*m/z* 499)(Table S1). The signal for cysteine (Cys) (*m/z* 120 as [Cys−H]^−^) was also observed in control of both cultivars (Table S1).

### Profiling pollen metabolites under heat conditions

**Figure 3:**
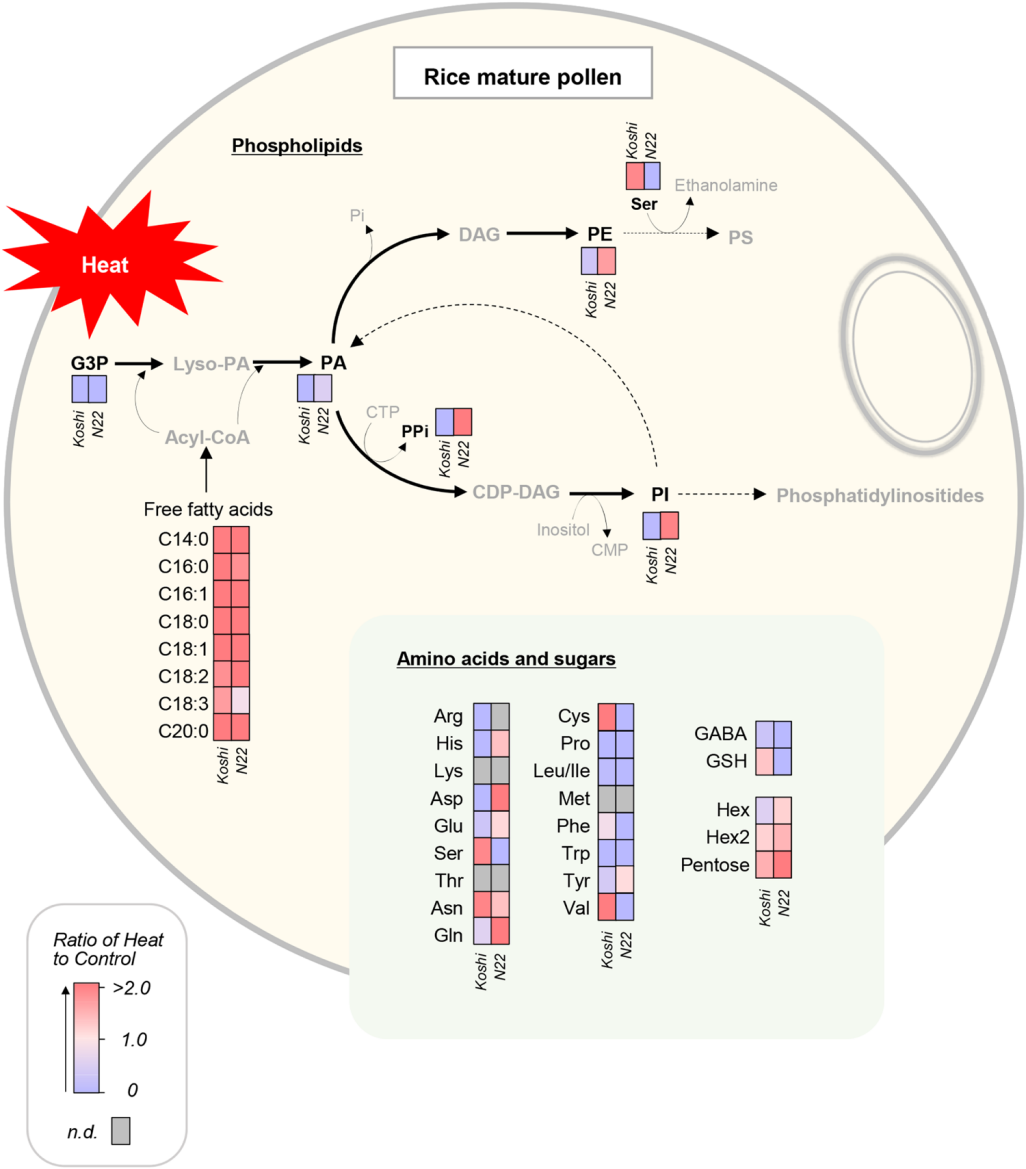
Putative phosphatidylinositol (C34:3) biosynthesis pathway and free fatty acids, amino acids, and sugars detected in the rice single mature pollen under heat conditions. The ratios of heat treatment to control in each cultivar are shown with a color scale. Trace amounts or absences of metabolites detected at less than 60% of frequency were shown in either gray letters and boxes. In each box set, Koshihikari and N22 are shown in left and right boxes, respectively.

When exposed to heat, the content of phospholipid synthesis-related metabolites, glycerol-3-phosphate and PA(34:3) declined in both cultivars (Table S1). However, the decline in PA(34:3) in N22 was much smaller than that of Koshihikari under heat conditions, and significant varietal difference was observed (*P* = 0.02). There were clear varietal differences in the accumulation pattern of the predominant PI(34:3) and PE(34:3) isomers in response to heat (Figs. 2 and 3, Table S1). Under the heat conditions, greater accumulation was observed in N22 for the predominant PI(34:3) and PE(34:3) isomers, but its content in Koshihikari was contrastingly declined. And consequently, there were significant varietal differences in heat-treated pollen at *P* = 0.13 and *P* = 0.21, respectively for the content of PI(34:3) and PE(34:3) isomers. The detected PI(34:3) isomer was shown to be composed of palmitoleic acid, C16:0 and linolenic acid, C18:3 by using MS/MS analysis (Fig. S2). PA(C36:6), PI(C36:6), and PE(C36:6) signals were less frequently detected in N22, compared with C34:3 species, but not for PC(36:6)(Table S1). In both cultivars, most fatty acids increased by heat, except for linolenic acid (*m/z* 277) in N22 (Fig. 2, Table S1). The ratio of C18:2 to C18:3 between control and heat treatment in Koshihikari changed little (4.25 and 3.94, respectively), although that in N22 dramatically declined from 13.67 down to 1.61.

Changes in these phospholipid signals including PI signals (C34:3 and C36:6) and their varietal differences were not similarly observed in the anther tissue samples (Fig. S1). There was little to no detection of phosphoinositide species, such as phosphatidylinositol-phosphate and phosphatidylinositol-bisphosphate, as well as other lipid-related metabolites including diacylglycerol (DAG), cytidine diphosphate (CDP), CDP-ethanolamine, PG(34:3), PIP3(34:3), PI(3)P(34:3), choline, CDP-choline (Citicoline), PIP2(34:3), and PIP2(36:6) in each treatment at the corresponding stage of development (see Table S1).

The content of Cys, serine (Ser)(*m/z* 104), and threonine (*m/z* 118) increased in heat-treated Koshihikari pollen grains, whereas serine (*m/z* 104) and threonine (*m/z* 118) signals declined substantially in heat-treated N22 pollen grains, but with little detection of Cys-related signals (*n* = 14, Table S1). Similar patterns were observed between cultivars for Cys-sugar cluster ions, such as [Cys+Hex−H]^−^ and [Cys+Hex_2_−H]^−^ (not detected and 7.1%, respectively in N22 pollen). The content of dehydroascorbic acid (*m/z* 173), oxaloacetic acid (*m/z* 131), citric acid (*m/z* 191), aspartic acid (*m/z* 132), glutamine (*m/z* 145), and methyl jasmonate (*m/z* 223) in Koshihikari decreased at high temperatures, whereas an increase in content of these metabolites and a cytokinin, presumably trans-zeatin (*m/z* 218) was observed in N22 (Table S1). For cell wall-related metabolites, the content of UDP (*m/z* 402), coumaryl-alcohol (*m/z* 149), and α-l-rhamnose (*m/z* 163) decreased in heat-treated Koshihikari, although those in N22 increased (Table S1).

## Discussion

**Figure 4:**
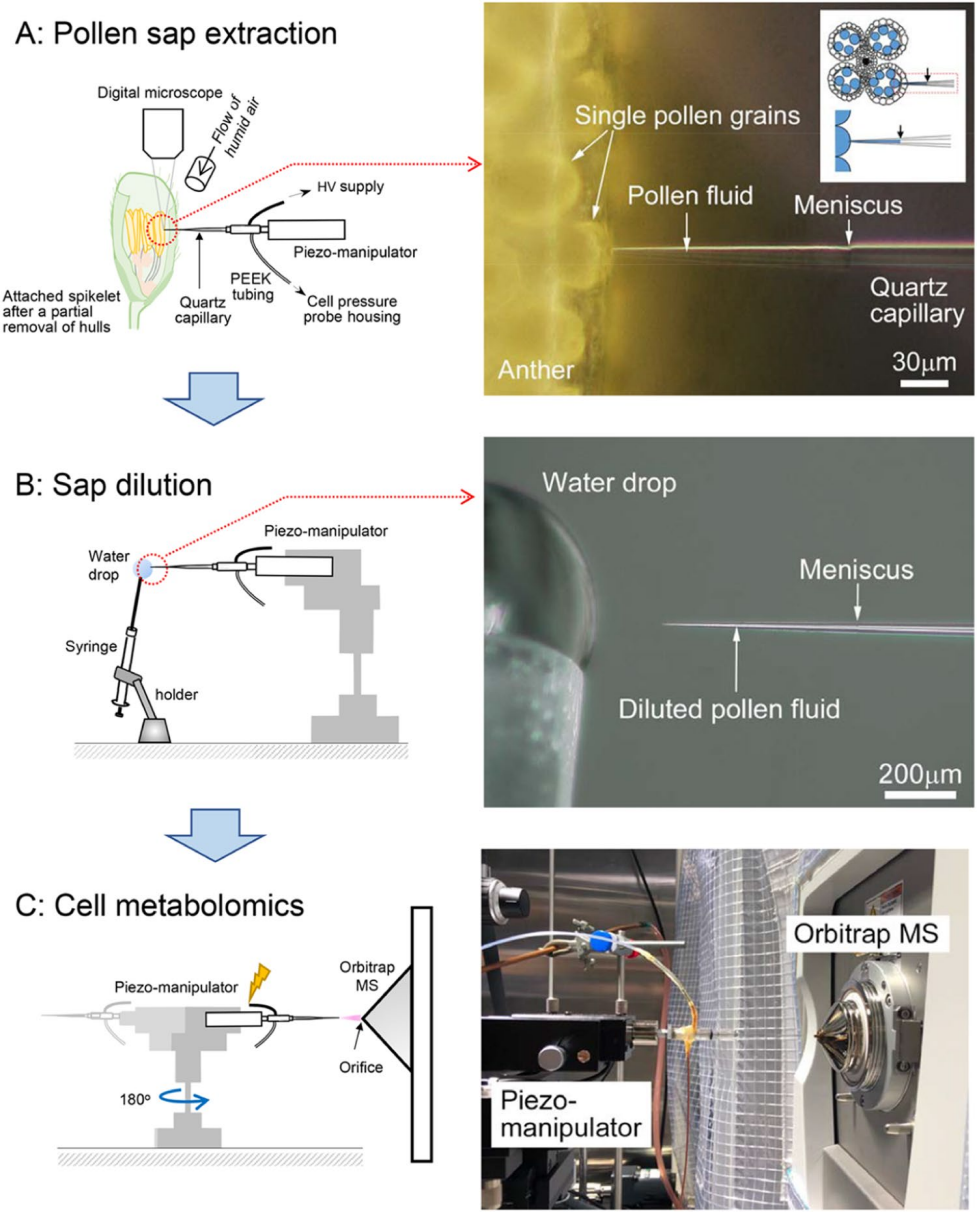
Illustrated workflow of on-site cell metabolomics in cellular fluids extracted from a single pollen grain located under the anther wall in intact plants which are exposed to heat conditions. The upper and lower insets in (**A**) shows a simple schematic of tip insertion into pollen on the anther cross section and anther side view, respectively. (**A**) shows a schematic of the fluid extraction and the microscopic image taken at the extraction. As soon as the cellular fluid was expelled into the capillary and the boundary (meniscus) was formed between the oil and cell sap, the tip was put into the water drop of ultrapure water for dilution (**B**). And then, the probe tip was immediately removed from the water drop and rotated 180° using a 3D move/rotation micro-manipulator. And instantly, the metabolites in the fluids were analyzed in Orbitrap mass spectrometer by applying a high voltage without any pre-treatment (**C**, see Methods).

In this work, we hypothesized that PI biosynthesis may be closely associated with heat tolerance in rice pollen for spikelet fertility. We collected picolitre pollen fluids from a growing single rice pollen grain to conduct on-site real-time metabolomics using picoPPESI-MS (Fig. 4). We have characterized changes in pollen metabolites in response to heat stress, as demonstrated by two cultivars exhibiting different spikelet fertility under heat conditions (Fig. 1A). At high temperatures, the content of PA declined in both cultivars and no phosphoinositide accumulation was observed at the mature stage of development; however, very clear and contrasting varietal differences in the accumulation of PI, composed of C16:0 and C18:3 (see Fig. S2), have been revealed prior to anther dehiscence and pollen germination (Figs. 2 and 3). Regardless of the application of heat treatment, heat tolerant cultivar, N22 exhibited greater PI accumulation than Koshihikari (Fig. 3), suggesting a close relation to maintaining high spikelet fertility (Fig. 4). Given into the fact that PI is a precursor of phosphoinositide species that induces multiple signaling as regulators of pollen hydration, germination, and tube growth^12,14–16^, we conclude that an active PI biosynthesis, detected at the single pollen grain level using picoPPESI-MS, is essential for male gametophyte development prior to the initiation of multiple signaling and contributes to heat tolerance for sustaining spikelet fertility under high temperature conditions.

Using a cell pressure probe, the direct turgor measurement has been made in a model plant, *Lilium longiflorum* pollen grains having typically 100 μm diameter^23^ and pollen tubes exhibiting between 100–1600 μm of tube length^24^. It seems that there have been no other pollen-related pressure probe works conducted in other species. In this work, we have directly inserted the microcapillary tip into growing rice pollen grains having 31–53 μm diameter (i.e., 15.3–77.4 pL)(see Fig. 4A), corresponding to 2.9–14.7% of *Lilium* pollen grain volume. Benkert *et al*. showed good pressure tracing over 15 min in lily pollen^24^. In the case of intact rice mature pollen examined here, it was possible to observe the rapid backward movement of oil/cell sap boundary (meniscus) in the microcapillary immediately after tip impalement (see Fig. 4A), indicating that pollen grains could exhibit positive turgor (i.e., internal hydraulic pressure) in mature pollen. However, it was not attainable to detect turgor accurately with a good stability or maintaining the meniscus location for long duration^25^, because of the relatively small pollen volume and occurrence of tip plugging during the meniscus oscillation due to the high viscosity of the pollen fluids in all treatments examined.

Recently, it has been pointed that most analyses conducted in the anthers or even flowers have been often regarded as the phenomenon confined to the pollen^26^. Despite of the impact on agricultural science, no attempts have been made for conducting metabolomics on pollen grains attached to growing crop plants. In this study, single pollen metabolomics using picoPPESI-MS has revealed the key pathway associated with heat tolerance (Figs. 2 and 3). Because of the high dilution rate (35-folds), it is postulated that the diluted fluids would be homogeneous in the capillary prior to electrospray ionization (Fig. 4C). The mass analyzer could quantify the amount of each metabolite ion in relation to the amount of the most abundant ion to give the relative abundance. Therefore, possible variations from the difference of dilution rate are likely to be ignorable as far as data analysis is on the relative abundance basis. Given into that considerable spatial heterogeneity in organelle arrangement occurs in mature rice pollen at polarity formation^10,27^, it is not surprising that slight differences in the insertion location of the target pollen grain might have caused some variations in MS spectra (see *p-*values in Table S1).

As well as other species, a series of physiological changes dramatically occurs in mature rice pollen prior to anther dehiscence and germination. As a result of the rapid decline in water content (86% down to 75%) accompanied by starch degradation, the concentration of sugars, amino acids, and inorganic phosphorous progressively increased under optimal temperature conditions^28^, although data on heat-induced pollen metabolisms, particularly internal lipids is lacking. In pooled wheat pollen, Narayanan *et al*. conducted lipidome analysis in heat-treated cultivars to report that C34:3 and C36:6 species dominated the composition of extraplastidic phospholipids including PI, although neither varietal differences in composition nor alteration of pollen lipidome was observed^29^. In this study, we have conducted single pollen metabolomics to increase the resolution, compared with conventional analysis. This allows us to find that there were clear varietal differences in heat responses on phospholipids, particularly for heat-induced accumulation of the predominant PI(34:3) and PE(34:3) in the heat-tolerant cultivar, N22 (Figs. 2 and 3, Table S1) that marked high spikelet fertility (Fig. 1A). The MS/MS data clearly showed that PI(34:3) is composed of C16:0 and C18:3 (Fig. S2). The secondary phospholipid C36:6 species was also detected (Table S1), consistent with ref. ^29^. It is expected that the C36:6 species in our pollen samples may have had two C18:3 chains. Different from other fatty acids increased under heat conditions (Fig. 3), a remarkable reduction for the heat-to-control ratio of C18:3 signal confined in N22 is very likely to refer to the active synthesis of the both PI(34:3) and PI(36:6) and other related phospholipids, as detected simultaneously (Table S1). Importantly, these PI-related signals were not observed in the anther tissue samples (Fig. S1), suggesting that spatial localization was confined to pollen grains. Aside from PI synthesis, C16 and C18 fatty acids identified here may also be used for pollen exine formation in rice^30^. Together with the above-mentioned lipids, it has been demonstrated that picoPPESI-MS is capable for detecting other metabolites, such as organic acids, amino acids, carbohydrates, cell wall-related materials, and hormones at the same time. These data indicate the robustness and usefulness of high-resolution *on-site cell-specific analysis* at single pollen level.

In general, mature pollen accumulate neutral lipids in lipid droplets, polar lipids in the form of densely packed membranes, and proteins required for tube growth^12^. Pollen lipid droplets are spherical organelles having a 0.5 to 2 μm diameter, which is smaller than the tip size (see Methods). And therefore, it is assumed that numerous lipid droplets and membranes in the vicinity of ER would have contaminated in the picolitre pollen fluid during the collection. Shintaku *et al*. have shown that single cell can be lysed by applying a bipolar voltage pulse (3 kV, 100 ms interval for each pulse)^31^. Regarding our picoPPESI-MS analysis, what exactly occurs during electrospray ionization remains to be investigated further in the view of analytical chemistry. However, given the fact that PI-like membrane phospholipids have been detected as strong signals in our analytical method, it is reasonably speculated that both lipid bodies and membrane that would be contaminated in our pollen fluid samples might be degraded in some manner when applying a high voltage (-4 kV) for ionization to occur. If this is the case, then the observed heat responses including the compositional changes in phospholipid metabolism would partially explain the spatial changes in organelle compartmentation that precedes to germination.

There is concrete evidence indicating that biosynthesis of phosphoinositide species activates to promote germination and polar tube growth through multiple signaling^12,14,15^. All phosphoinositide species could derive from PI by phosphorylation of the lipid head group and are generated by one or two phosphorylation steps with phosphates catalyzing the reverse reactions. Therefore, significant PI accumulation observed in heat-treated N22 pollen could facilitate the forthcoming multiple signaling for pollen tube growth to succeed in double fertilization, resulting in the superior spikelet fertility observed in Fig. 4A. The reduction in PA (34:3) content and greater heat-induced pyrophosphate accumulation in N22 may support an activation of PI synthesis (Fig. 3). It is also anticipated that either activation in PI synthase^32^ and/or the reduction in PI-specific phospholipase C activity^33,34^ may be the key enzymes regulating phospholipid metabolism to cope with heat. Alternative explanation is that considerable differences in PI turnover rate might exist between cultivars (Fig. 3). In N22, the rate of PI turnover might have slowed down to hinder phosphoinositide signaling from turning on pollen germination prior to anther dehiscence. If Koshihikari pollen had rapid PI turnover rate under heat conditions, PI might be degraded by phospholipase D to PA transformed into any other glycerophospholipid^35^. If this holds true, it is possible to interpret that in heat susceptible cultivars’ pollen phospholipase D may be activated by heat. Any mutant of the PI synthase-encoding genes has not been reported yet^12^; however, our data strongly suggest that active biosynthesis and accumulation of the major PI (C34:3) is essential in the ungerminated rice pollen and is closely associated with spikelet fertility under heat conditions. Based on our finding, it is suggested that quantity and composition of PI may be used as an alternative method for evaluating pollen viability.

There is no direct evidence that increases in ROS scavenging activity sustains pollen development under heat conditions^26^. Pollen is known to accumulate numerous mitochondria, twenty times more than in vegetative cells^36^, and therefore pollen mitochondria may be the main source organelle of ROS produced during pollen development. In the present study conducted in single pollen, we have observed contrasting responses between cultivars in terms of TCA cycle metabolites, OAA, succinic, fumaric, and citric acids in response to heat (Table S1), suggesting that varietal difference in systematic ROS regulation may exist in pollen mitochondria. In this study, ROS signals, such as H_2_O_2_ themselves were undetectable because they were below the detection limit of Orbitrap MS (i.e., *m/z* < 50); however, accumulation pattern for several redox metabolites, ascorbic acid and glutathione, consistently observed in all treatments (see control in Table S1), implies that certain ROS concentrations may be necessary for pollen maturation. In high night temperature-treated sorghum plants, it has been reported that changes in membrane-associated phospholipids accompanied by increasing ROS may be responsible for the reduction in pollen functions^17^. Likewise, there may be a close relation between PI-related synthesis and ROS concentration in rice pollen, which remains unexplored. However, it is noteworthy that pollen size was sustained under heat conditions in both cultivars (Fig. 4), presumably by osmotic adjustment as discussed below. And, it should be emphasized that opposite responses to heat for sugars, redox metabolites and Cys-related signals were observed between two cultivars (see Results, Table S1).

In general, Cys helps in the stabilization of the protein structure through the formation of disulfide bonds in the cells. More recently, we have used the same analytical method to report that cytosolic Cys accumulation occurred in heat-treated rice endosperms that reduced protein synthesis rate prior to chalky formation^18^. Similarly in rice pollen, our data is suggestive that heat tolerant rice cultivar pollen may have an effective mechanism for facilitating biosynthesis of proteins, such as phospholipid pathway-related enzymes and heat-shock proteins^26^, leading to the optimal pollen development prior to germination under heat conditions. If carbohydrate starvation were ongoing in heat-treated Koshihikari pollen, cytosolic Cys would contribute to increase in osmotic pressure (i.e., osmotic adjustment) to maintain pollen volume (Fig. 1B), presumably through a partial protein synthesis inhibition. Hence, the observed varietal differences in Cys-related signals may reflect the differences in the synthesis rate of proteins needed for germination. In this view, it is presumed that N22 pollen would be more effectively adjusted osmotically, compared with Koshihikari.

In conclusion, we have shown that the synthesis of a phospholipid, PI, directly detected in the mature rice pollen under heat conditions may be important for multiple signaling leading to polar tube growth after adhesion to the stigma and contribute to heat tolerance, maintaining spikelet fertility. We propose that PI biosynthesis may serve as an alternative biomarker for evaluating pollen viability, instead of conventional *in vitro* pollen viability using iodine staining. Further analysis may shed additional light on enhancing rice heat tolerance through the identification of regulatory gene(s) to high temperature conditions. As cell heterogeneity has been increasingly recognized as a biologically important phenomenon observable in plants, conducting cell metabolomics including picoPPESI-MS analysis may open new possibilities to reveal many more cell-specific physiological events in greater depth than more conventional tissue-level analysis.

## Methods

### Plant material

A growth-chamber experiment was conducted at the Kyushu Okinawa Agricultural Research Center, Chikugo, Japan. Rice seeds from two cultivars, a heat-susceptible cultivar, *Oryza sativa* L. ‘Koshihikari’^22^ and heat-tolerant cultivar, N22^21^ were provided. Each plant was grown in a plastic pot, as described previously^37^. They were grown by removing the tillers periodically to restrict each plant to its main culm to minimize sample-to-sample vaiations^22,38^ and cultivated in a cycle of day/night air temperatures of 26 °C (13 h, 5:50–18:50)/22 °C (11 h, 18:50–5:50) at 60% Relative humidity (RH) until heading to rule out the potential effects of the history of heat conditions prior to heading. Two treatments were applied at heading: no heat as control (26 °C) and high temperature (34 °C). For the high-temperature treatment, pots were transferred to another growth chamber set at 34/28 °C and 70/80% RH at 9:00 on the day of heading. The photosynthetically active radiation (PAR) and the photoperiod were the same as the first growth chamber (750 μmol m^−2^ s^−1^ and 13/11 h day/night, respectively). After 48 h of high-temperature treatment, the pots were transferred back to the control chamber to grow until they reached the mature stage of plants (40 days after heading; DAH). In total there were 14 pots in control and 16 pots in the heat treatment were prepared for each cultivar.

### On-site cell metabolomics

By using picoPPESI-MS^19^, on-site cell metabolomics was conducted on single pollen grains of intact plants growing under heat conditions in the growth chambers (K260B029-S01, Tsubuku Corporation Ltd., Kurume, Japan), as reported in the previous study (see Fig. S1 in ref. ^18^). A potted plant at the 2^nd^ day of heat treatment, corresponding to maturation stage of pollen grains^10^, was placed at the center of a U-shaped vibration-free table in the measurement room (see Fig. S1 in ref. ^18^). The one of the first to third primary rachis branches, counted from the top of the intact panicle (i.e., the upper position in the panicle), were fixed on the sample holder with magnets, and then the top half of the lemma in the attached superior spikelets was quickly removed under humid conditions, and the attached anthers were gently fixed on the sample holder with surgical tape. Using a Piezo manipulator (DC-3K, Märzhäuser Wetzlar, Germany), the tip of microcapillary filled with a 0.01% (v/v) ionic liquid/silicone oil mixture^19^ was inserted into the developing pollen grains adjacent to the inner wall of the middle theca (Fig. 4A) to collect cellular fluid (4.7 ± 3.0 pL [mean ± SD, *n* = 21]). Immediately after the probe tip was moved into a water drop (*r* = ca. 0.7 mm), and the fluids were diluted to avoid tip plugging at ionization by instantly reducing the oil pressure to sub-zero pressure (Fig. 4B). The probe tip was rotated and oriented toward the opening of an Orbitrap mass spectrometer (Q-Exactive, ThermoFisher Scientific Inc., MA, USA), and the tip was subsequently charged at −4 kV using a high-voltage generator (AKTB-05k1PN/S, Touwa Keisoku Corp., Tokyo, Japan) (Fig. 4C). The MS analysis was then conducted. Spikelets attached to the sample holder were humidified throughout the process, and all analyses were confined to superior spikelets in the upper position of panicle and the entire process of picoPPESI-MS analysis was completed within a few minutes per shot. Although humid conditions induced anther dehiscence, no septum rupture occurred during the above analysis. All manipulations were conducted under a digital microscope (KH-8700, HIROX Co. Ltd., Tokyo, Japan). The mass spectra reported here are representative of the repeated measurements on 8–16 pollen grains from 4–5 independent plants in each treatment.

### Identification of phosphatidylinositol and anther sample analysis

Exact monoisotopic *m/z* values for all the peaks on the mass spectra acquired were extracted using the Qual Browser application in the Thermo Xcalibur software (ThermoFisher Scientific). Metabolites were identified from the theoretical masses of candidate metabolites in the METLIN online metabolomics database (http://metlin.scripps.edu/index.php) allowing differences of <5 ppm. In addition to the single pollen analyses described above, MS/MS analysis for PI(34:3) was conducted on N22 pollen grains in intact plants, together with standard l-α-PI sodium salt from *Glycine max* (Sigma-Aldrich). Collision-induced dissociation (CID) tandem MS analysis was performed using the same Orbitrap MS coupled with the picoPPESI system, which allowed us the MS/MS identification of target molecules in the same picolitre pollen fluids. The MS scan was performed in negative ion mode with the same instrument settings as described above, except that the resolution was 70000. Additionally, MS analysis was conducted on the crude anther extracts collected from three plants in each treatment for both cultivars at the same stage. The anthers from two superior spikelets attached to the same position in a panicle were collected using forceps, frozen at -80 °C for >2 h, and freeze-dried. The sample was then mixed with 50% (v/v) water/methanol and sonicated. After centrifugation for 10 min at 10 000 *g* at 4 °C, the supernatant (i.e. crude tissue extract) was used for tissue MS analyses. All the standard chemicals and organic solvents used in the experiments, except for the standard PI from *Glycine max*, were LC/MS grade purchased from Wako Pure Chemical Industries, Ltd. (Osaka, Japan). Ultrapure water of 18.2 MΩ cm^−1^ was used throughout the experiment.

### Microscopy

By disrupting the developing anther samples from the superior spikelets attached to the same upper position in a panicle used for picoPPESI analysis, ungerminated mature pollen was collected. Pollen was viewed and photographed after being stained in potassium iodide for 1 min, and *in vitro* pollen viability was determined. The anther length and pollen grain diameter on the light microscopic images were determined using ImageJ software (US National Institutes of Health). The values reported for pollen grain diameter represent the means of 3–6 independent plants (37–212 pollen grains per plant). For pollen number per anther and anther length, three plants were used in each treatment.

### Spikelet fertility

After harvest, the panicles were dried at 30 °C for 3 days. Thereafter, the number of mature seeds and empty caryopses was counted. The values reported for spikelet fertility represent the means of the spikelets attached to the same position from 3–4 independent plants (i.e., panicles) in each treatment.

### Statistical analysis

For spikelet fertility, anther length, pollen grain, and *in vitro* pollen viability, data were subjected to two-way ANOVA (cultivar × treatment) to assess the effects of cultivar and treatment and their interaction on each parameter in JMP (version 12.1.0; SAS Institute Inc., Cary, NC, USA). Significant differences between means were then determined using the Tukey’s test at 95% confidence (*P* ≤ 0.05). Analysis of all metabolome data was performed using Student’s *t* test in JMP.

## Electronic supplementary material

supplementary material.

supplementary material2.

## Data Availability

The datasets generated during and/or analyzed during the current study are available from the corresponding author on reasonable request.
