# Supplementary material for: On-site single pollen metabolomics reveals varietal differences in phosphatidylinositol synthesis under heat stress conditions in rice
Source: Sci Rep. 2020 Feb 6;10:2013. doi: 10.1038/s41598-020-58869-9 (PMC7005239; doi:10.1038/s41598-020-58869-9)
Supplement: Supplementary file 1 — supplementary material. [file 41598_2020_58869_MOESM1_ESM.doc]

**Corresponding author:** Hiroshi Wada (ORCID ID 0000-0003-0510-5744; Phone +81-942-52-0670; FAX +81-942-53-7776; Email: [hwada@affrc.go.jp](mailto:hwada@affrc.go.jp))

Article type: Original Research Article

Title: On-site single pollen metabolomics reveals varietal differences in phosphatidylinositol synthesis under heat stress conditions in rice

**Authors:** Hiroshi Wada^1,†,*^, Yuto Hatakeyama^1,†^, Taiken Nakashima^2^, Hiroshi Nonami^3^, Rosa Erra-Balsells^4^, Makoto Hakata^1^, Keisuke Nakata^3^, Kenzo Hiraoka^5^, Yayoi Onda^3^, and Hiroshi Nakano^1^

**Affiliations:** ^1^Kyushu Okinawa Agricultural Research Center, National Agriculture and Food Research Organization, 496 Izumi, Chikugo, Fukuoka 833-0041, Japan

^2^Faculty of Agriculture, Hokkaido University, Kita-9 Nishi-9, Kita-Ku, Sapporo, 060-8589, Japan

^3^Graduate School of Agriculture, Ehime University, 3-5-7 Tarumi, Matsuyama, 790-8566, Japan

^4^Department of Organic Chemistry, University of Buenos Aires, Buenos Aires 1428, Argentina

^5^Clean Energy Research Center, The University of Yamanashi, 4-3-11 Takeda, Kofu, Yamanashi 400-8511, Japan

^†^These authors contributed equally to this work.

ORCID ID; Yuto Hatakeyama (0000-0003-0526-4091), Taiken Nakashima (0000-0002-9384-7993), Hiroshi Nonami (0000-0002-4336-9049), Rosa Erra-Balsells (0000-0003-0169-0173), Makoto Hakata (0000-0001-9771-4103), Keisuke Nakata (0000-0003-3767-1089), Kenzo Hiraoka (0000-0002-5352-5914), Yayoi Onda (0000-0003-1398-3635), and Hiroshi Nakano (0000-0002-0564-2550)

*E-mail Yuto Hatakeyama: hatakeyamay067@affrc.go.jp, Taiken Nakashima: tnak005@res.agr.hokudai.ac.jp, Hiroshi Nonami: nonami@agr.ehime-u.ac.jp, Rosa Erra-Balsells: erra@qo.fcen.uba.ar, Makoto Hakata: hakata@affrc.go.jp, Keisuke Nakata: k.nakata.1013@gmail.com, Kenzo Hiraoka: hiraoka@yamanashi.ac.jp, Yayoi Onda: onda.yayoi.ui@ehime-u.ac.jp, and Hiroshi Nakano: nakanohr@affrc.go.jp*

Submission date: October 21, 2019

Figure. S1


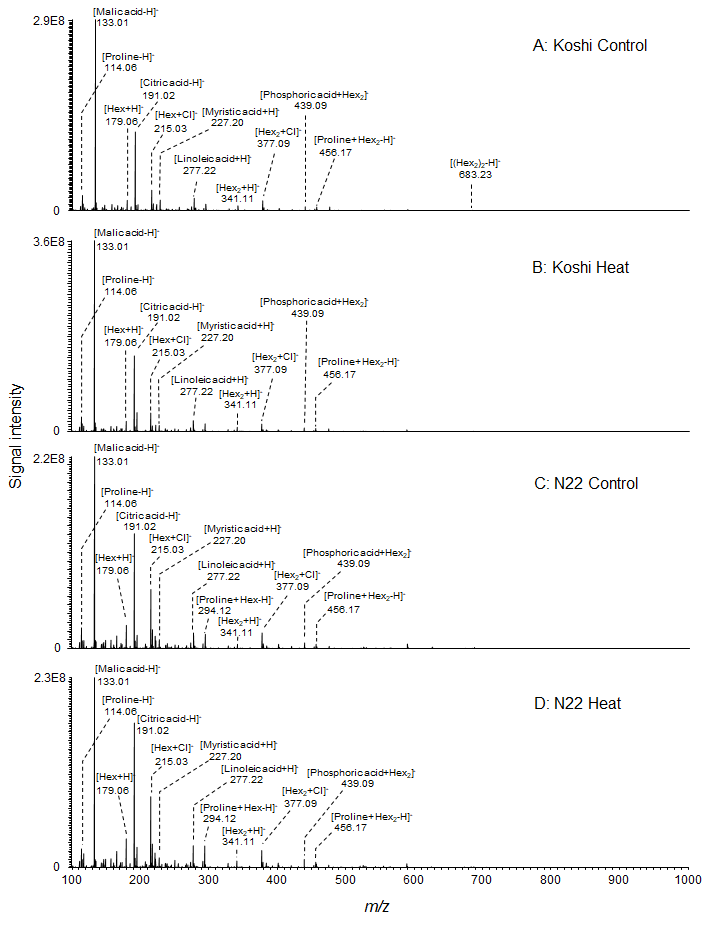


Figure S1. Mass spectra for picoPPESI-MS in negative ion mode obtained from the anther tissue extracts for the different treatments. The data are representative of repeated experiments with anther tissue extracts from 3 plants in each treatment.

Figure S2.


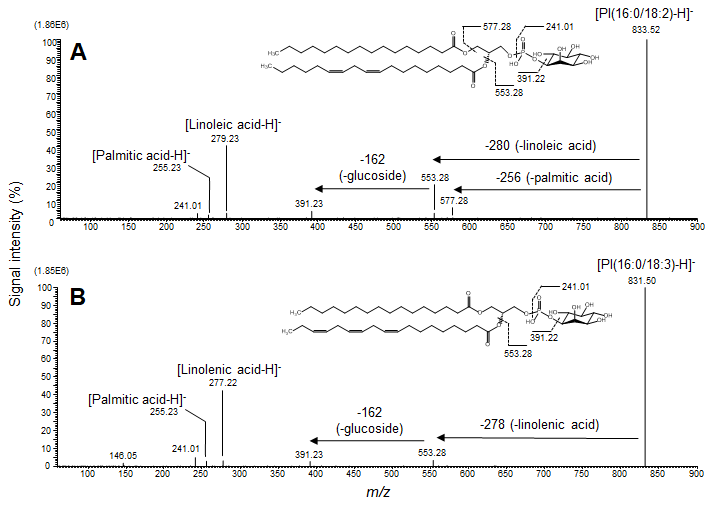


Fig. S2. (A) PicoPPESI-MS/MS spectrum of the standard L-α-phosphatidylinositol (PI) (16:0/18:2)([M-H]^-^, *m/z* 833.52) solution from *Glycine max* in negative ion mode*.* Prior to the analysis, the standard solution was diluted 100-fold (chloroform/methanol/distilled water)(70/27/3, v/v). Selector gate range was *m/z* 833.3-833.7. (B) PicoPPESI-MS/MS spectrum of putative PI (16:0/18:3) ([M-H]^-^, *m/z* 831.50) signal obtained from pollen sap in negative ion mode. Precursor ion *m/z* 831.50; selector gate range *m/z* 831.3-831.7. In both analyses, normalized collision energy was set to be 20 %, and HCD fragmentation ions were detected in the Orbitrap at a resolution setting of 140,000. According to the MS/MS spectrum, the precursor ion in B was identified as [PI (16:0/18:3)-H]^-^.
